# Supplementary material for: Carcinoid Syndrome and Hyperinsulinemic Hypoglycemia Associated with Neuroendocrine Neoplasms: A Critical Review on Clinical and Pharmacological Management
Source: Pharmaceuticals (Basel). 2021 Jun 4;14(6):539. doi: 10.3390/ph14060539 (PMC8228616; doi:10.3390/ph14060539)
Supplement: Supplementary file 1 [file pharmaceuticals-14-00539-s001.zip › pharmaceuticals-1207548-supplementary.pdf]

Table S1. Different vasoactive substances implicated in the pathogenesis of clinical symptoms in carcinoid syndrome**.**

**Substance**	**Associated symptoms**
Serotonin (5-HT)	Diarrhoea, flushing, palpitations
Tachykinins	Diarrhoea, flushing, palpitations
Histamine	Flushing, wheezing, palpitations
Kallikrein	Flushing, palpitations
Prostaglandin	Diarrhoea, flushing, palpitations
Catecholamines	Flushing, palpitations
Motilin	Diarrhoea
